# Supplementary material for: Cardiac fibroblasts regulate the development of heart failure via Htra3-TGF-β-IGFBP7 axis
Source: Nat Commun. 2022 Jun 7;13:3275. doi: 10.1038/s41467-022-30630-y (PMC9174232; doi:10.1038/s41467-022-30630-y)
Supplement: Supplementary file 1 — Supplementary Information [file 41467_2022_30630_MOESM1_ESM.pdf]

Supplementary Information

Cardiac fibroblasts regulate the development of heart failure

via Htra3-TGF-β-IGFBP7 axis

Supplementary Fig. 1

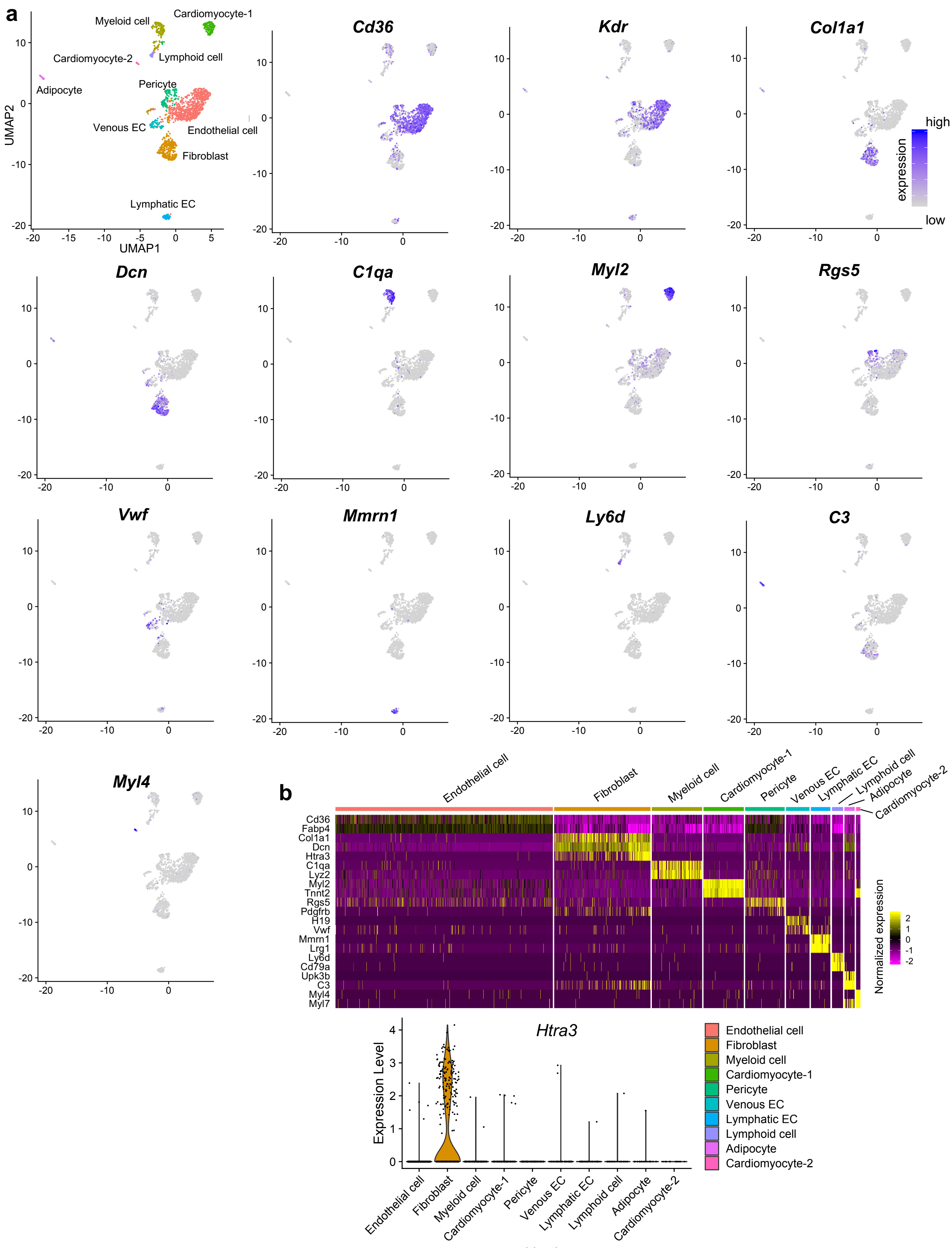

Supplementary Fig. 1

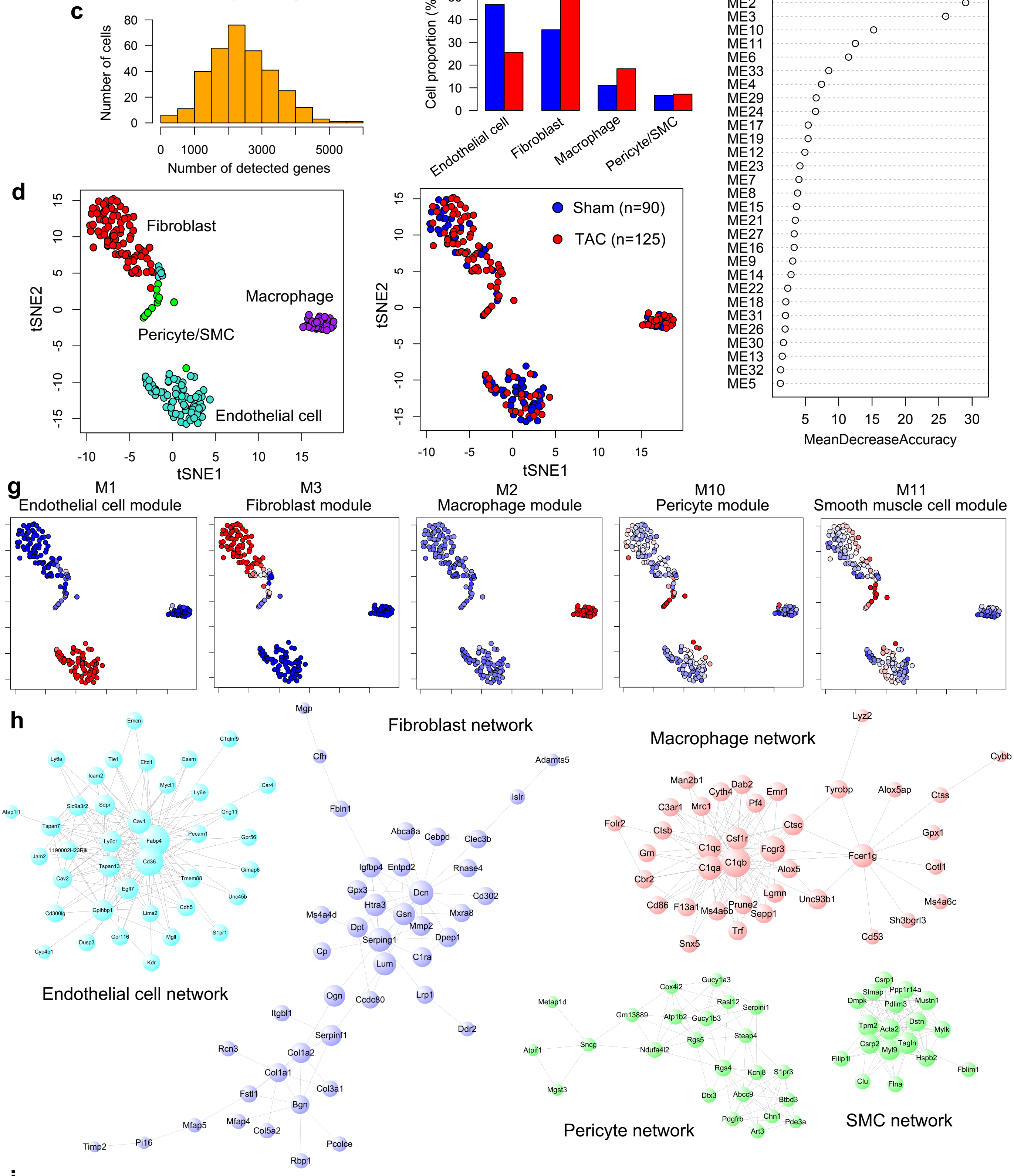

i

Endothelial cell module

Enriched Term	PValue
Membrane	7.03E-24
Glycoprotein	2.34E-10
Tetraspanin	5.06E-08
Cell-cell adherens junction	2.89E-07

Fibroblast module

Enriched Term	P-Value
Extracellular matrix	4.07E-44
Secreted	5.77E-42
Collagen fibril organization	1.31E-13
Endoplasmic reticulum	2.20E-10

Macrophage module

Enriched Term	P-Value
Lysosome	1.19E-23
Chemotaxis	2.62E-14
Immunity	1.59E-13
Glycoprotein	4.33E-13

Pericyte/SMC module

Enriched Term	P-Value
Vascular smooth muscle contraction	7.07E-06
Mitochondrion	3.32E-05
Cell-cell adherens junction	4.02E-05
Focal adhesion	7.49E-05

j

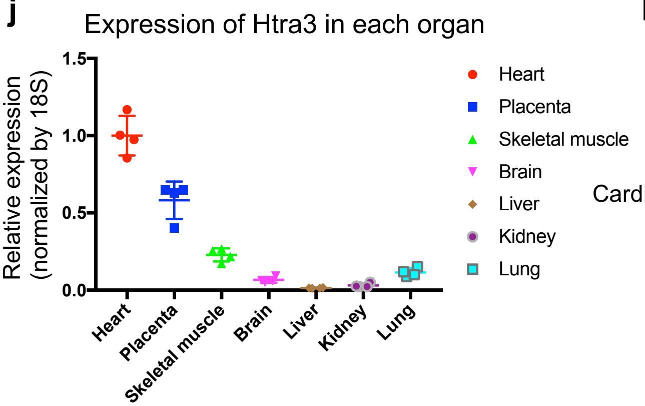

k

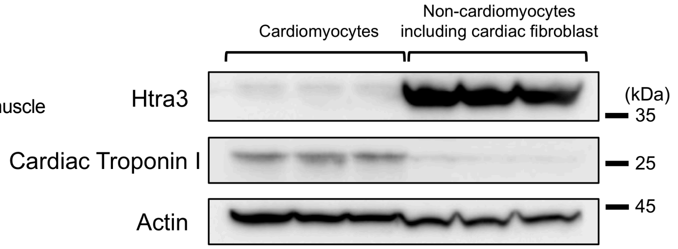

Supplementary Fig. 1 | Single-cell RNA-seq of non-cardiomyocytes in the heart using the Chromium (10X Genomics) and Smart-seq2 platform, related to Fig. 1

a, Uniform Manifold Approximation and Projection (UMAP) plot of single-cell transcriptomes of non-cardiomyocytes (n = 1,783) from the murine heart (n = 2). Cell-type–specific expression profiles are also shown.
b, Heatmap indicating the cell-type-specific expression profiles of genes including *Htra3* (upper). Violin plot showing the fibroblast specific expression of *Htra3* (lower).
c, Histogram showing the distribution of the number of detected genes. Cells in which over 2,000 genes were detected were used for subsequent analysis.
d, T-distributed Stochastic Neighbor Embedding (tSNE) plot of single-cell transcriptomes of non-cardiomyocytes (n = 90 for sham and n = 125 for transverse aortic constriction [TAC]) from the murine heart (n = 2 for sham and n = 2 for TAC). Cell type was determined by graph-based clustering and random forest classifier (left). The origin of the cells is also annotated (right).
e, Bar graph showing the distribution of cell types in sham (blue) and TAC surgery (red).
f, Mean decrease in accuracy (in order of decreasing accuracy from top to bottom) of each module eigengene (ME) as assigned by the random forest classifier.
g, tSNE plot showing each ME expression. Module names were annotated by cell-type–specific expression detected by each module network in **(h)**.
h, Co-expression network of each module. The size of the dots represents node centrality.
i, Gene ontology (GO) analysis of each module. The most characteristic GO terms in annotation clusters that ranked in the top 4 in the “Functional Annotation Clustering” function with statistical significance (P < 0.05) were extracted for each module. *p*-values are determined by Fisher's Exact test.
j, mRNA expression levels of *Htra3* in various organs of mice were assessed by real-time qPCR (n = 4 at each organ, respectively). Data are shown as mean and SD. Source data are provided as a Source Data file.
k, Western blot of Htra3 using cardiomyocytes and non-cardiomyocytes including cardiac fibroblasts.

Supplementary Fig. 2

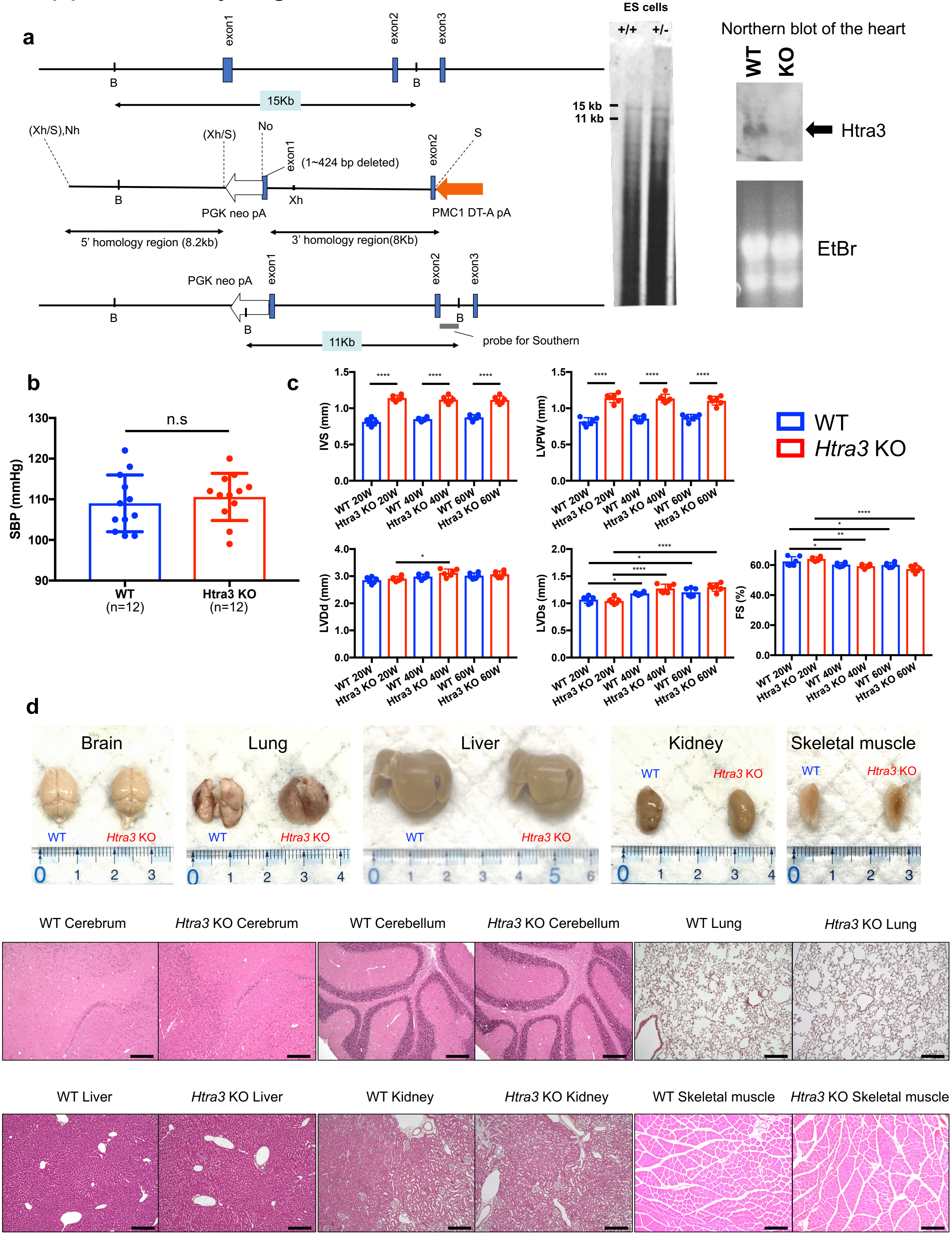

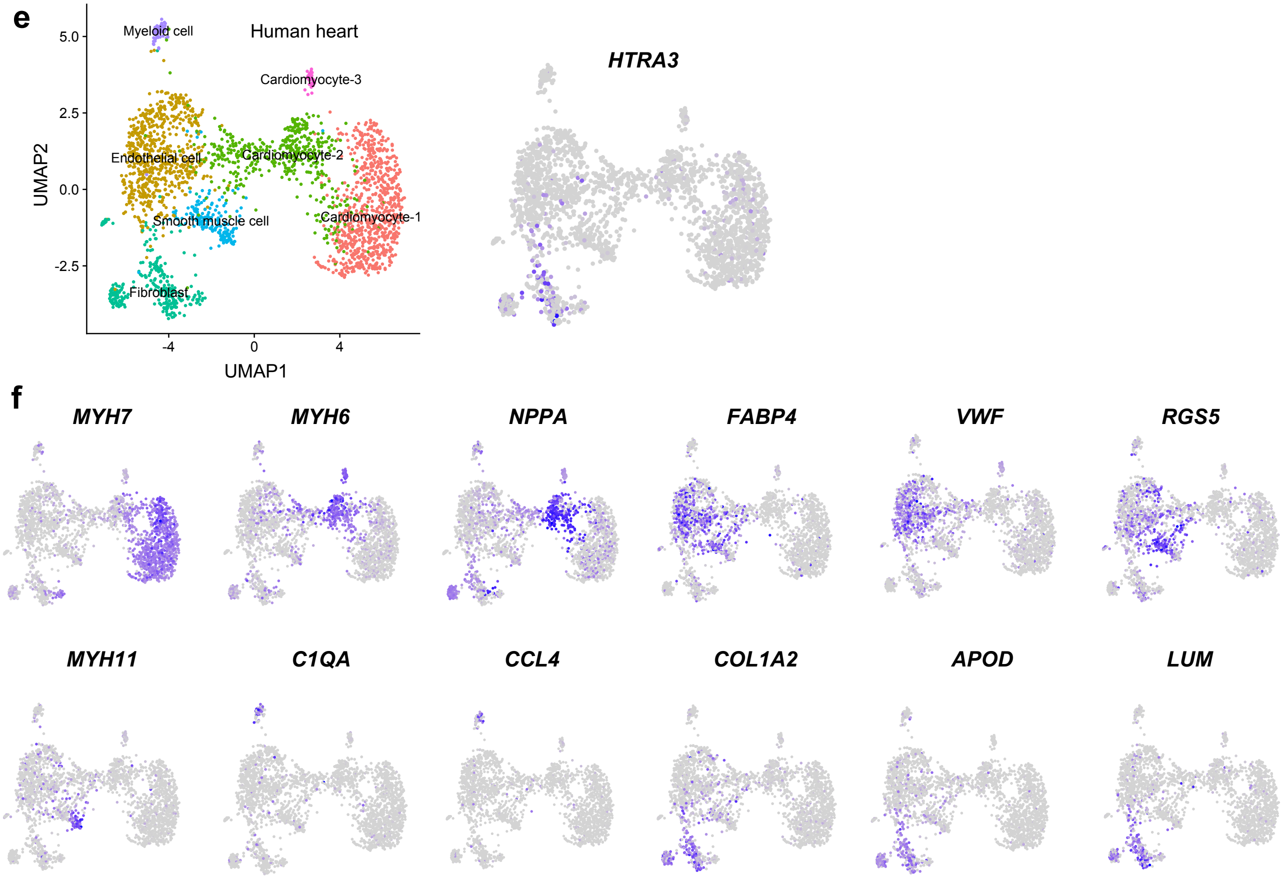

Supplementary Fig. 2 | Generation of *Htra3* KO mice and single-cell RNA-seq showing the *HTRA3* expression in the human heart, related to Fig. 2

a, Genome structure of mouse *Htra3* (upper), targeting vector (middle), and targeted *Htra3* gene (lower). The 5'-end of Htra3 exon1 (1-424 bp) was deleted. Genomic DNA from targeted ES cells were digested with Bam HI and subjected to Southern blot analysis. The DIG-labeled probe (derived from intron 2 indicated as gray line) detected a 15-kb fragment from the WT *Htra3* allele and a 11-kb fragment from the targeted allele. Northern blot of total RNA prepared from wild-type (WT) and homozygous *Htra3* knockout (KO) mice shows the absence of the *Htra3* transcript in heart. B, BamHI site; S, Sal I site; Xh, Xho I site; No, Not I site. Open arrow shows a PGK-neoPolyA cassette. Orange arrow shows a PMC-diphtheria toxin (DT-A)-PolyA cassette.
b, Comparison of systolic blood pressure of WT and *Htra3* KO mice (n = 12, respectively). Data are presented as mean values +/- SEM. Significance was determined by an unpaired two-tailed Student's t-test. Source data are provided as a Source Data file.
c, Echocardiographic assessment of the heart from WT and *Htra3* KO mice in different stages of age (n = 6, respectively). Data are shown as mean and SD. Significance was determined by one-way ANOVA with Tukey's or Dunnett's post hoc test. * P < 0.05, ** P < 0.01, **** P < 0.001. Source data are provided as a Source Data file.
d, Morphological and histological comparison of multiple organs between WT and *Htra3* KO mouse. Scale bar, 100 μm
e, UMAP plot of scRNA-seq profiles of the human heart (N=2,459 cells). Expression levels of *HTRA3* and *LUM* are also shown.
f, Cell-type-specific expression profiles on the UMAP plot.

Supplementary Fig. 3

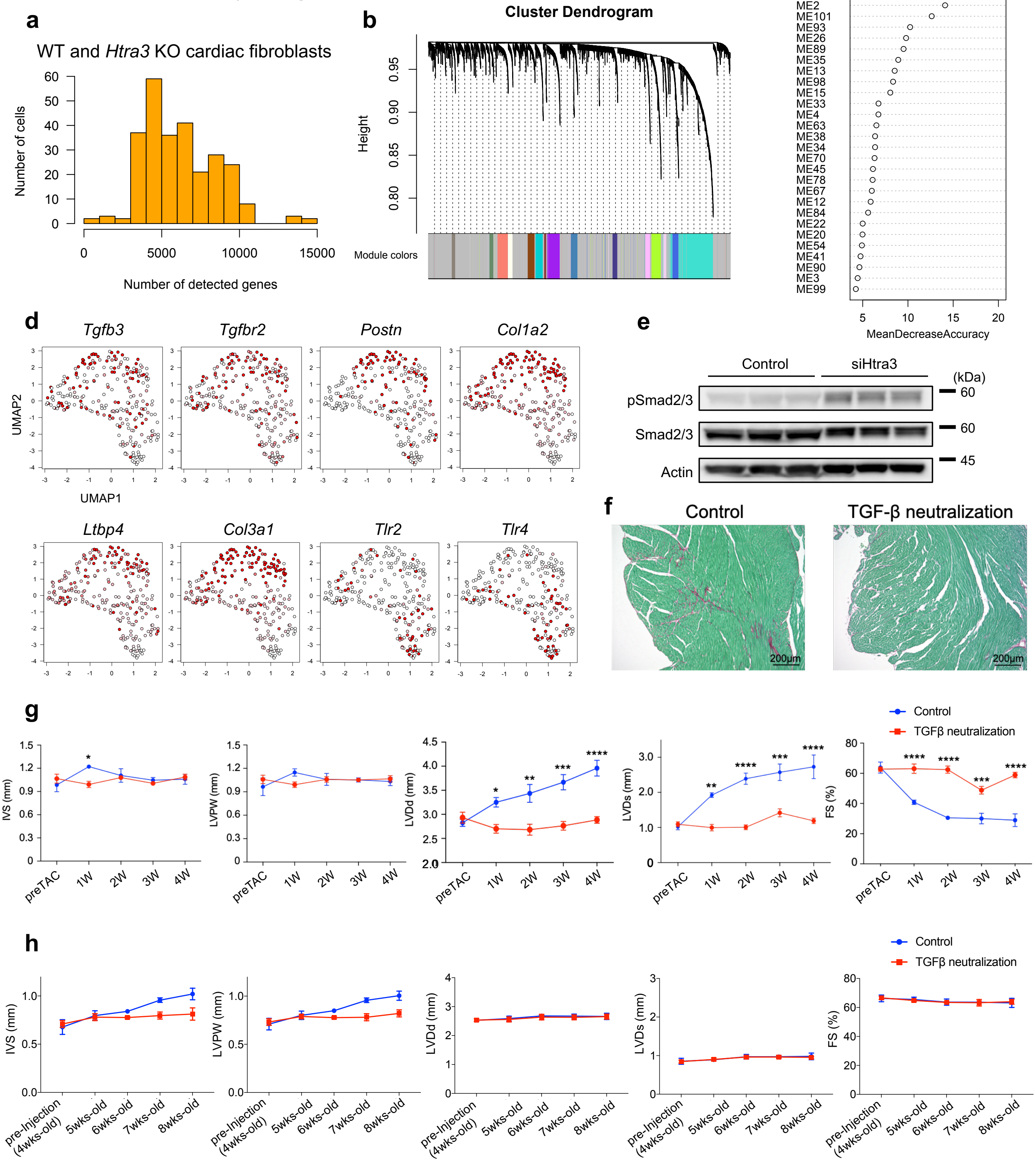

Supplementary Fig. 3 | Single-cell RNA-seq analysis of cardiac fibroblasts and the effects of TGF-β neutralization in the TAC model, related to Fig. 3

a, Histogram showing the number of detected genes in scRNA-seq of cardiac fibroblasts from WT and *Htra3* KO mice. Single-cell transcriptomes in which over 3,000 genes were detected were used for subsequent analysis.
b, Dendrogram showing the clustered modules assigned using weighted gene correlation network analysis (WGCNA).
c, Mean decrease in accuracy (in order of decreasing accuracy from top to bottom) of each ME as assigned by the random forest classifier.
d, Module-specific expression profiles on the UMAP plot.
e, Western blot analysis using cardiac fibroblasts. TGF-β1 treatment was used as a positive control for pSmad2/3.
f, Histochemical detection of collagen fibres by Sirius Red/Fast Green dye staining in WT and *Htra3* KO mice at 4 weeks after TAC with or without TGF-β neutralization antibody treatment.
g, Echocardiographic assessment of the heart of *Htra3* KO mice after TAC with or without TGF-β neutralization antibody treatment (n = 3 each). Data are shown as mean and SD. * $P < 0.05$, ** $P < 0.01$, *** $P < 0.005$, **** $P < 0.001$; significance was determined by two-way ANOVA with Bonferroni's multiple comparison test. Source data are provided as a Source Data file.
h, Echocardiographic assessment of the heart of *Htra3* KO mice after injection of TGFβ antibody or IgG isotype control antibody (n = 3 vs 3). Data are shown as mean and SD. Significance was determined by two-way analysis of variance (ANOVA) with Bonferroni's multiple comparison test. ** P < 0.01, *** P < 0.005, and **** P < 0.001. Source data are provided as a Source Data file.

Supplementary Fig. 4

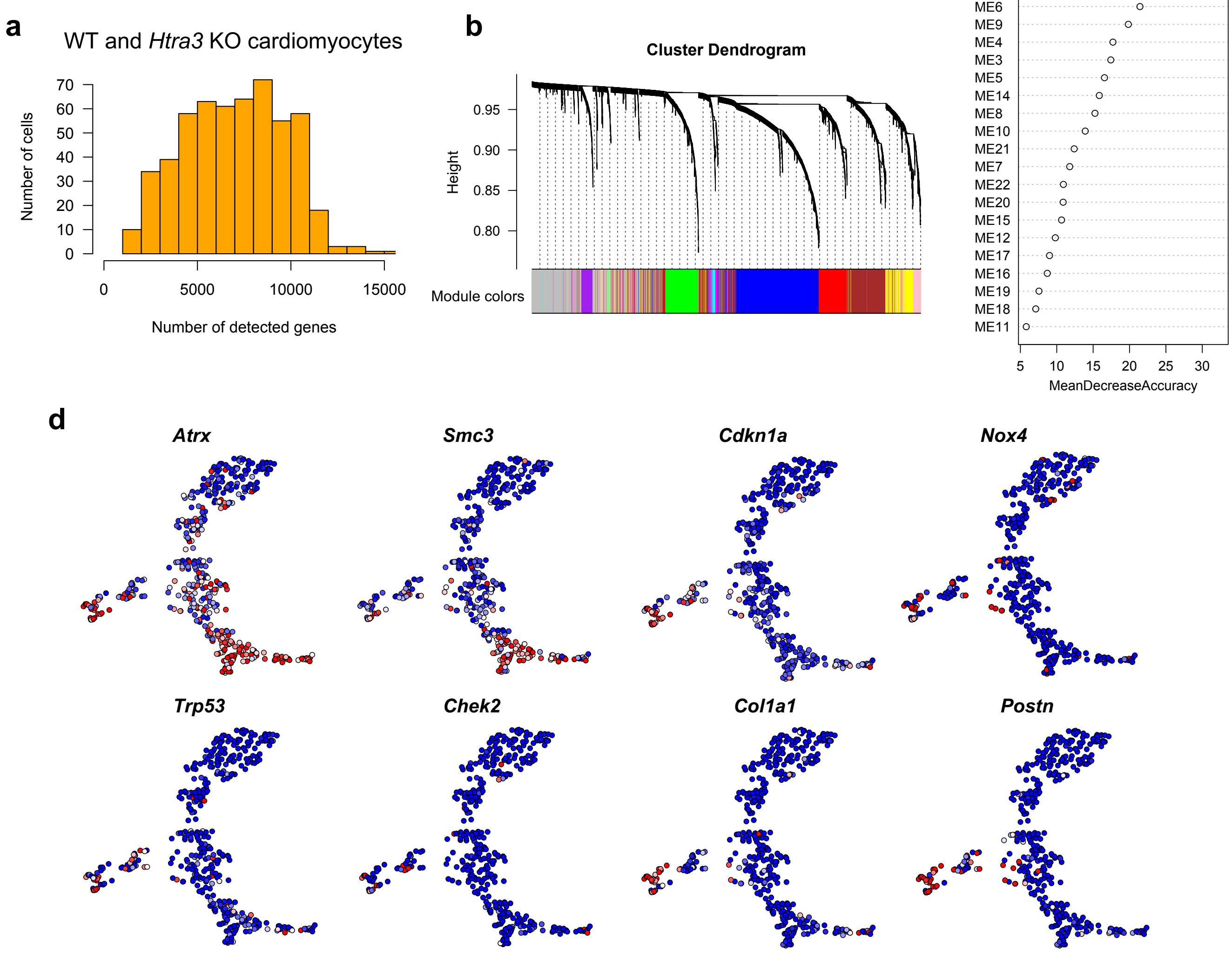

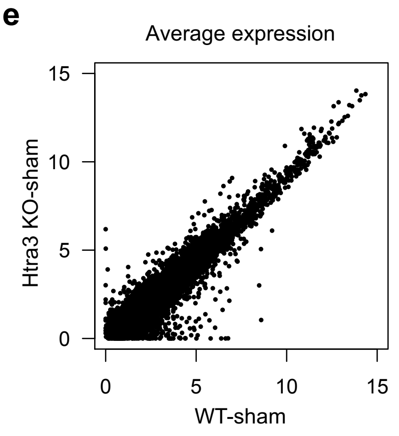

Up-regulated in Htra3 KO-sham
Translation (*P*=5.44e-9)
Cellular response to hypoxia (P=2.87e-4)
Protein stabilization (P=1.05e-3)
Down-regulated in Htra3 KO-sham
Oxidation-reduction process (P=6.63e-10)
Glutathione metabolic process (P=3.96e-6)
Reactive oxygen species metabolic process (P=1.09e-5)

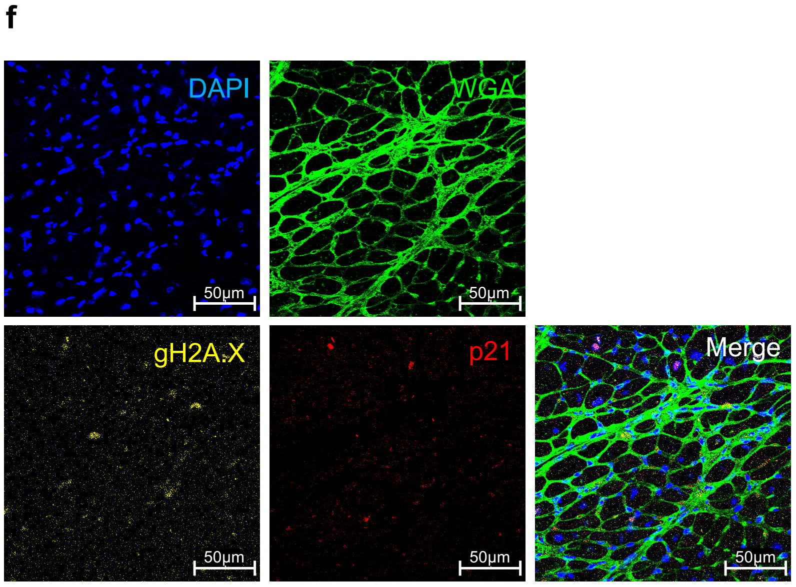

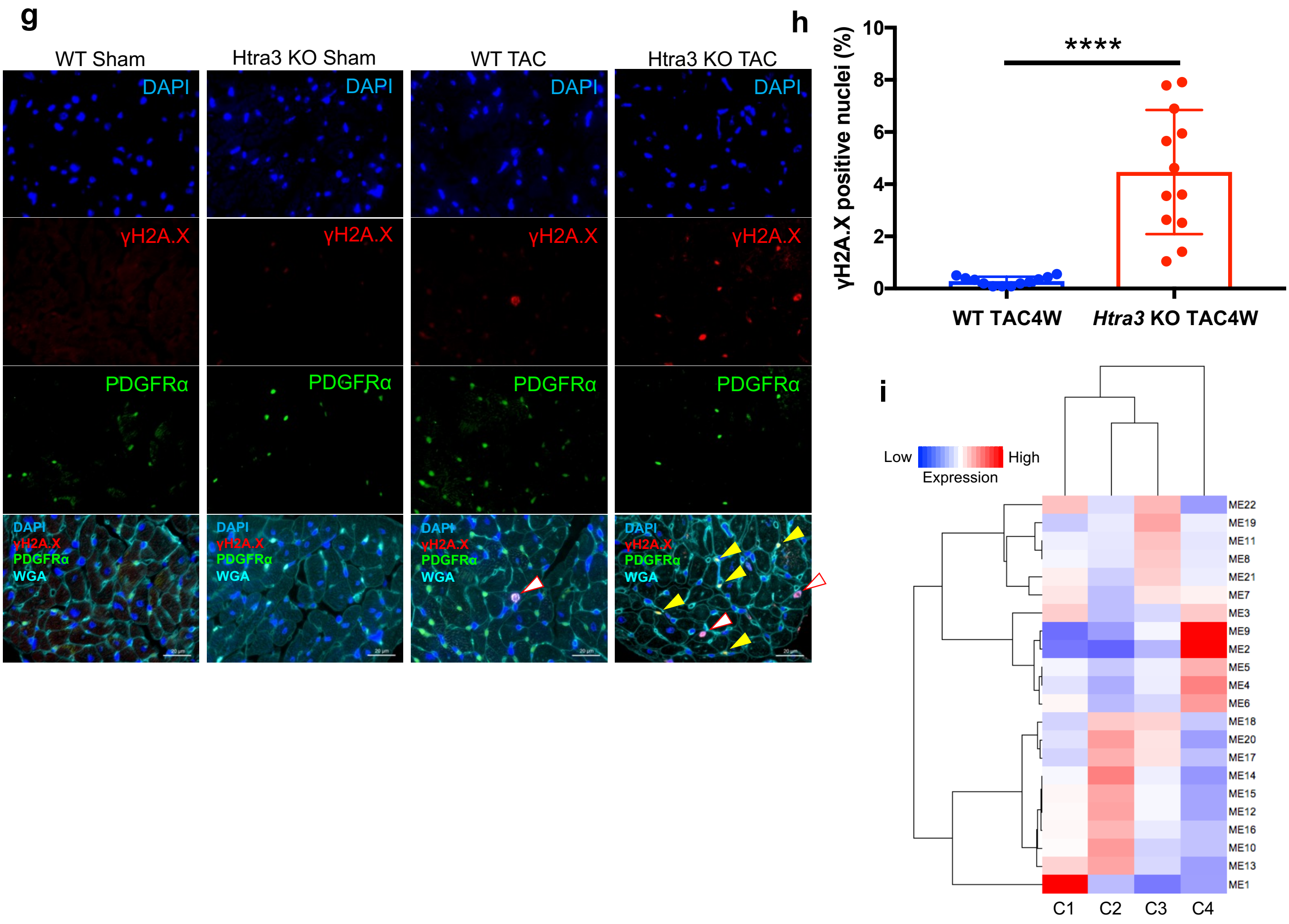
g
WT Sham
Htra3 KO Sham
WT TAC
Htra3 KO TAC
DAPI
γH2A.X
PDGFRα
WGA
h
γH2A.X positive nuclei (%)
0
2
4
6
8
10
****
WT TAC4W
Htra3 KO TAC4W
i
Low
High
Expression
ME22
ME19
ME11
ME8
ME21
ME7
ME3
ME9
ME2
ME5
ME4
ME6
ME18
ME20
ME17
ME14
ME15
ME12
ME16
ME10
ME13
ME1
C1
C2
C3
C4

Supplementary Fig. 4 | Single-cell RNA-seq analysis of cardiomyocytes from WT and *Htra3* KO mice, related to Fig. 4

a, Histogram showing the number of detected genes in scRNA-seq of cardiomyocytes from WT and *Htra3* KO mice. Single-cell transcriptomes in which over 4,000 genes were detected were used for subsequent analysis.
b, Dendrogram showing the clustered modules assigned using WGCNA.
c, Mean decrease in accuracy (in order of decreasing accuracy from top to bottom) of each ME as assigned by the random forest classifier.
d, Module-specific expression profiles on the UMAP plot.
e, Scatter plot showing the average expression profiles of scRNA-seq of isolated cardiomyocytes from WT and *Htra3* KO mice after sham surgery. Enriched GO terms of top 300 upregulated and downregulated genes and associated P-values are also shown. *p*-values are determined by Fisher's Exact test. Upregulation of genes associated with translation in *Htra3* KO cardiomyocytes suggests the transcriptional signature of hypertrophied cardiomyocytes in *Htra3* KO mice.
f, Immunostaining of γH2A.X (Yellow) and p21 (Red) on heart sections from *Htra3* KO mice 4 weeks after TAC surgery. WGA (Green) and DAPI (Blue) are used to stain the plasma membrane and nucleus, respectively.
g, Immunostaining of gH2A.X (Red) and PDGFRa (Green) on heart sections from WT and *Htra3* KO mice after TAC or sham surgery (4 weeks). Yellow arrows indicate the γH2A.X/PDGFRa-positive cardiac fibroblasts, while white arrows indicate the gH2A.X-positive cardiomyocytes.
h, Quantification of γH2A.X positive nuclei in the hearts of WT and *Htra3* KO mice after TAC surgery (n = 12, respectively). Data are presented as mean values +/- SEM. **** $P < 0.001$; Significance was determined by an unpaired two-tailed Student’s t-test. Source data are provided as a Source Data file.
i, Hierarchical clustering of ME expression.

Supplementary Fig. 5

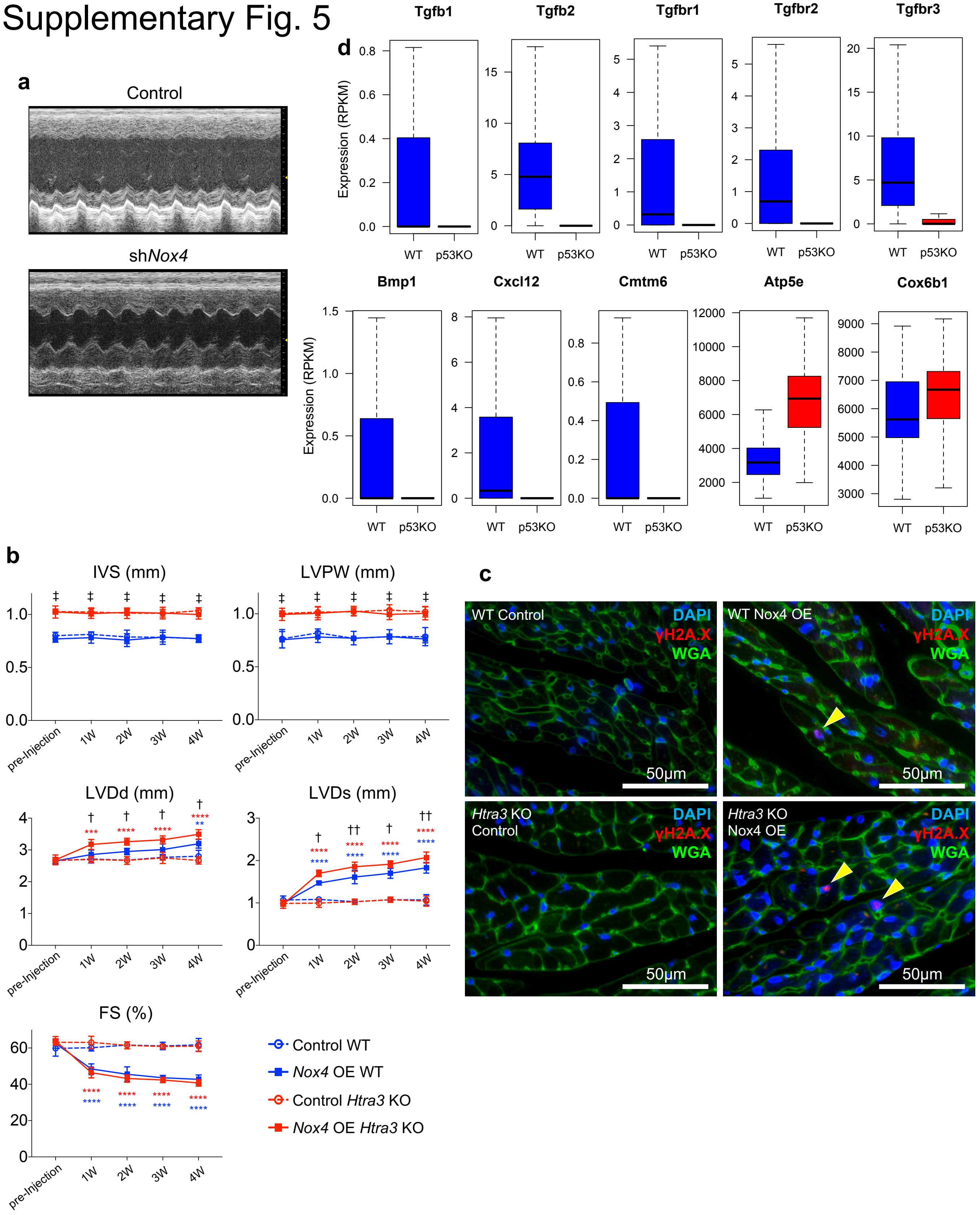

Supplementary Fig. 5 | Functional and transcriptional analysis of sh*Nox4*-induced mice and p53 KO mice, related to Fig. 5

a, Representative echocardiographic images of shControl- or sh*Nox4*-induced mice 4 weeks after TAC surgery.
b, Echocardiographic assessment of the heart of WT and *Htra3* KO mice after infection of the AAV9-Nox4 overexpression (OE) vector or the Control vector. n = 3 (WT Control), n = 5 (WT Nox4 OE), n = 3 (Htra3 KO Control), n = 5 (Htra3 KO Nox4 OE). Data are shown as mean and SD. ** P < 0.01, *** P < 0.005, and **** P < 0.001 Nox4 OE vs Control (Blue for WT, Red for Htra3 KO), † P < 0.05, and †† P < 0.01 WT Nox4 OE vs Htra3 KO Nox4 OE, ‡ P < 0.001 Htra3 KO (both Nox4 OE and Control) vs WT (both Nox4 OE and Control); significance was determined by two-way analysis of variance (ANOVA) with Bonferroni‘s multiple comparison test. Source data are provided as a Source Data file.
c, Immunostaining of γH2A.X (Red) on heart sections from WT and *Htra3* KO mice after TAC or sham surgery (4 weeks). Yellow arrows indicate the γH2A.X-positive cardiomyocytes.
d, Boxplot showing expression profiles of TGF-β signaling molecules, secretory factors, and mitochondrial genes in cardiomyocytes isolated from WT and cardiomyocyte-specific p53 KO mice at 2 weeks after TAC (n = 48 cells for WT, n = 48 for p53KO). Single-cell RNA-seq data was from Nomura, S. et al. Cardiomyocyte gene programs encoding morphological and functional signatures in cardiac hypertrophy and failure. Nat. Commun. 9, 4435 (2018). Data represent box plots and individual data points. Box plots show the median (center line), first and third quartiles (box edges), while the whiskers going from each quartile to the minimum or maximum.

Supplementary Fig. 6

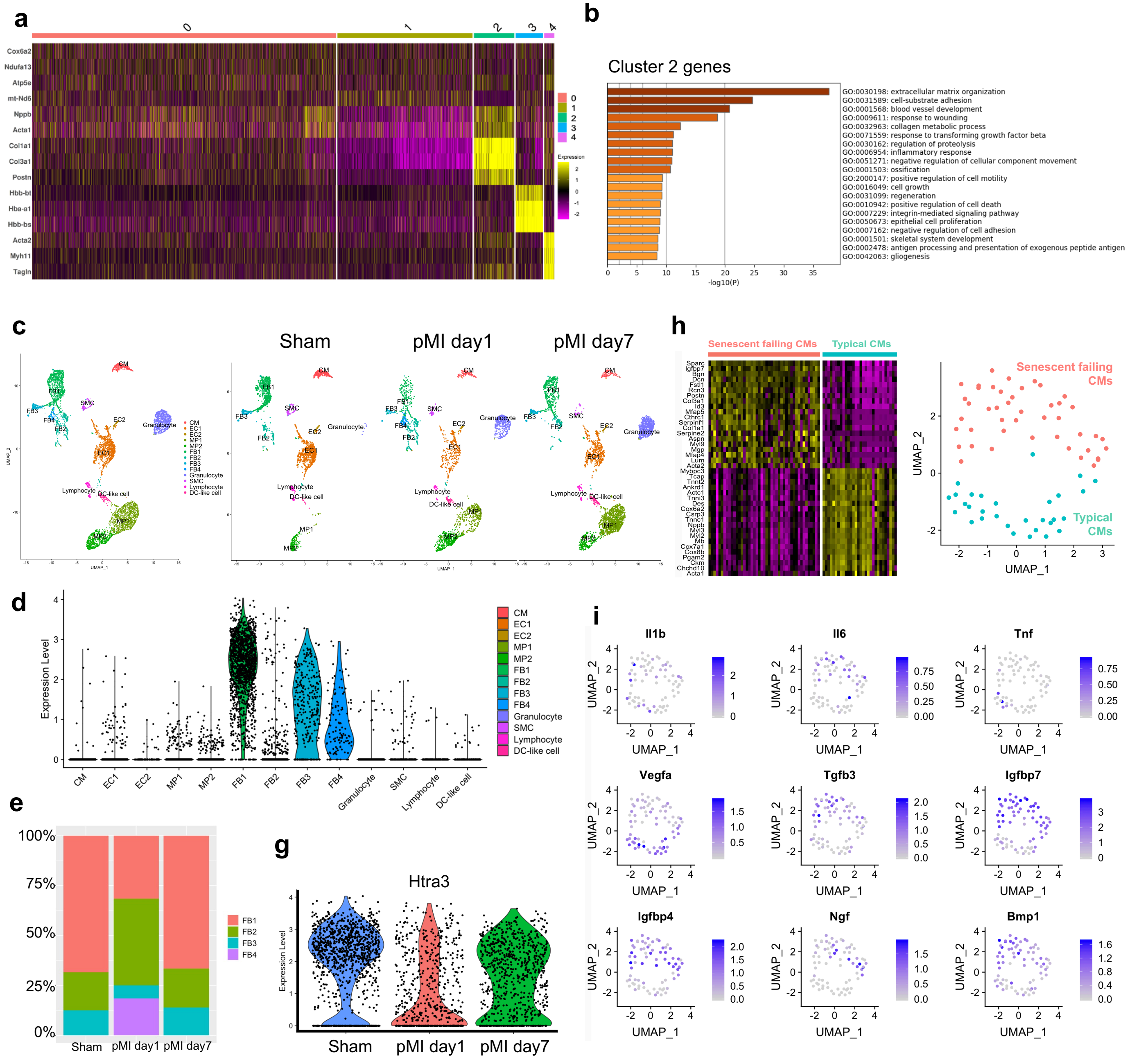

f

GO analysis of FB2 characteristic genes

	P-Value	Benjamini score
Translation	2.90E-41	4.30E-38
rRNA processing	8.00E-11	6.00E-08
Negative regulation of apoptotic process	6.60E-10	3.30E-07
Cytoplasmic translation	1.40E-09	5.20E-07
Ribosomal small subunit assembly	1.60E-08	5.00E-06
Ribosomal large subunit biogenesis	4.70E-08	1.20E-05
Ribosomal small subunit biogenesis	2.60E-07	5.70E-05
Ribosomal large subunit assembly	2.00E-06	3.90E-04
Protein folding	4.00E-06	6.70E-04
Cell-cell adhesion	6.40E-06	9.70E-04

GO analysis of FB4 characteristic genes

	P-Value	Benjamini score
Cell adhesion	2.70E-19	3.50E-16
Collagen fibril organization	3.20E-19	3.50E-16
Wound healing	9.70E-09	7.10E-06
Cell migration	5.90E-08	3.30E-05
Heart development	1.50E-07	6.60E-05
Endodermal cell differentiation	1.90E-07	7.10E-05
Skeletal system development	3.90E-07	1.20E-04
Protein folding	6.40E-07	1.80E-04

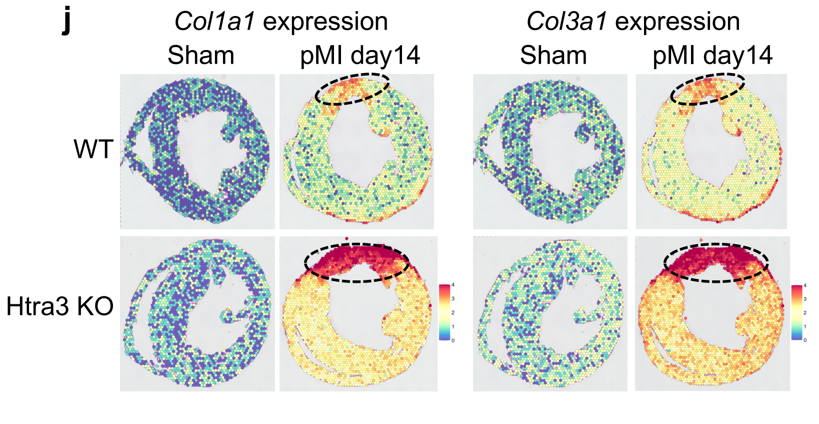

Supplementary Fig. 6 | Spatial transcriptomic analysis and single-cell RNA-seq of cardiomyocytes after MI shows Htra3 repression-mediated spatial induction of senescent failing cardiomyocytes, related to Fig. 6

a, Heatmap showing the expression levels of representative genes of each cluster.
b, Enriched GO terms and enrichment P-values associated with the representative genes of cluster 2.
c, UMAP plot of single-cell transcriptomes of non-cardiomyocytes from the sham and infarcted heart (n = 9,029 cells; Sham 2,673 cells, pMI day 1 2,580 cells, pMI day 7 3,776 cells). CM, cardiomyocyte; EC, endothelial cell; MP; macrophage; FB, fibroblast; SMC, smooth muscle cell; DC, dendritic cell.
d, Heatmap showing the expression patterns of genes characteristic for senescent failing cardiomyocytes and adaptive cardiomyocytes isolated from the infarct zone of mice at 7 days after MI operation. UMAP plot of total cells are also shown.
e, Expression patterns of characteristic genes on the UMAP plot.
f, Violin plot showing *Htra3* RNA expression in each cell cluster in sham, post MI (pMI) day 1, and pMI day 7.
g, Bar plot showing the distribution of FB clusters in sham, post MI (pMI) day 1, and pMI day 7.
h, Total expression level of *Htra3* in sham, post MI (pMI) day 1, and pMI day 7.
i, Enriched GO terms of differentially expressed genes seen in FB2 and FB4 cluster. *p*-values are determined by Fisher's Exact test.
j, The spatial expression patterns of genes characteristic for senescent failing cardiomyocytes.

Supplementary Fig. 7

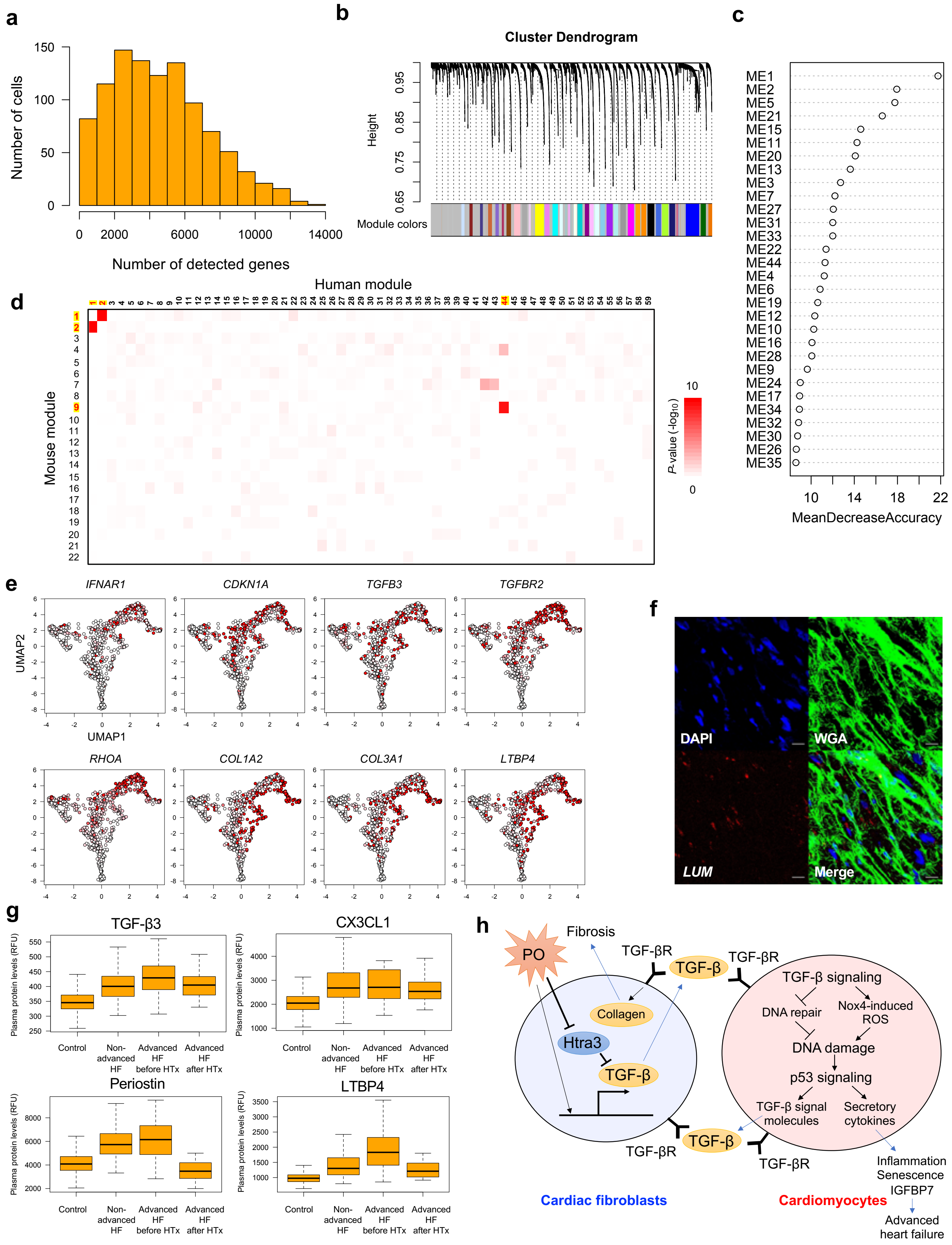

Supplementary Fig. 7 | Single-cardiomyocyte RNA-seq and plasma proteome analysis of patients with heart failure, related to Fig. 7

a, Histogram showing the number of detected genes in scRNA-seq of cardiomyocytes from control subjects (n = 2) and patients with heart failure (n = 22). Single-cell transcriptomes in which over 2,000 genes were detected were used for subsequent analysis.
b, Dendrogram showing the clustered modules assigned using WGCNA.
c, Mean decrease in accuracy (in order of decreasing accuracy from top to bottom) of each ME as assigned by the random forest classifier.
d, Cross-tabulation table of cardiomyocyte modules obtained from mouse scRNA-seq (Fig. 3 and Supplementary Fig. 7) and from human scRNA-seq (Fig. 4 and Supplementary Fig. 9). The table is colored by $-\log_{10}$ (*P*-value), obtained using two-sided Fisher's exact test. Modules colored yellow and red are significantly conserved (all module enrichment *P*-values were $>1\times10^{-5}$).
e, Module-specific expression profiles on the UMAP plot.
f, Single-molecule RNA *in situ* hybridization of *LUM* in cardiac tissue from patients with heart failure. Scale bar, 20 μm
g, Boxplot of plasma protein levels of secretory cytokines. RFU, relative fluorescence unit. HF, heart failure. HTx, heart transplantation. Data represent box plots and individual data points. Box plots show the median (center line), first and third quartiles (box edges), while the whiskers going from each quartile to the minimum or maximum. n = 768 for Control, n = 85 for non-advanced HF, n = 30 for advanced HF before HTx, n = 30 for advanced HF after HTx.
h, Schematic of the mechanism by which Htra3 in cardiac fibroblasts prevents TGF-β signaling-induced cardiomyocyte senescence and heart failure. PO, pressure overload.
