# Supplementary material for: Cardiac fibroblasts regulate the development of heart failure via Htra3-TGF-β-IGFBP7 axis
Source: Nat Commun. 2022 Jun 7;13:3275. doi: 10.1038/s41467-022-30630-y (PMC9174232; doi:10.1038/s41467-022-30630-y)
Supplement: Supplementary file 3 — Reporting Summary [file 41467_2022_30630_MOESM3_ESM.pdf]

nature research

Corresponding author(s): Issei Komuro

Last updated by author(s): Mar 4, 2022

Reporting Summary

Nature Research wishes to improve the reproducibility of the work that we publish. This form provides structure for consistency and transparency in reporting. For further information on Nature Research policies, see our Editorial Policies and the Editorial Policy Checklist.

Statistics

For all statistical analyses, confirm that the following items are present in the figure legend, table legend, main text, or Methods section.

n/a	Confirmed	
☐	☒	The exact sample size (*n*) for each experimental group/condition, given as a discrete number and unit of measurement
☐	☒	A statement on whether measurements were taken from distinct samples or whether the same sample was measured repeatedly
☐	☒	The statistical test(s) used AND whether they are one- or two-sided<br>*Only common tests should be described solely by name; describe more complex techniques in the Methods section.*
☒	☐	A description of all covariates tested
☒	☐	A description of any assumptions or corrections, such as tests of normality and adjustment for multiple comparisons
☐	☒	A full description of the statistical parameters including central tendency (e.g. means) or other basic estimates (e.g. regression coefficient) AND variation (e.g. standard deviation) or associated estimates of uncertainty (e.g. confidence intervals)
☐	☒	For null hypothesis testing, the test statistic (e.g. *F*, *t*, *r*) with confidence intervals, effect sizes, degrees of freedom and *P* value noted<br>*Give P values as exact values whenever suitable.*
☒	☐	For Bayesian analysis, information on the choice of priors and Markov chain Monte Carlo settings
☒	☐	For hierarchical and complex designs, identification of the appropriate level for tests and full reporting of outcomes
☐	☒	Estimates of effect sizes (e.g. Cohen's *d*, Pearson's *r*), indicating how they were calculated

Our web collection on statistics for biologists contains articles on many of the points above.

Software and code

Policy information about availability of computer code

Data collection	Microscope: LSM 880 META confocal microscope (Zeiss), BZ-X700 microscope (Keyence), In Cell Analyzer 6000 (GE Healthcare). Echocardiography: Vevo 2100 imaging system (Visualsonics, Inc.). Flow cytometory: FACSJazz cell sorter (BD Biosciences, the version of software is ver1.2). qPCR: CFX96 Real-Time PCR Detection System (Bio-Rad). DNA electrophoresis: LabChip GX (Perkin Elmer), TapeStation 2200 (Agilent Technologies). Western blot: LAS 4000 analyser (GE Healthcare).
Data analysis	Single-cell RNA-seq analysis: R (v3.5.3), Tophat (v2.1.1), Cufflinks (v2.2.1), Bowtie (v1.1.1), DEGseq (v1.8.0), WGCNA (v1.6.8), Cytoscape (v3.7.2), scran (v1.16.0), Rtsne (v0.15), umap (v0,2,6,0), Slingshot (v1.6.1), Cluster 3.0, JAVA Treeview (v1.1.6r4), randomForest (v4.6-14), Cell Ranger (v3.0.2), Seurat (v3.1.4). Imaging analysis: BZ Analyzer software (v1.1.1.8, Keyence). Statistical analysis: R (v3.5.3), GraphPad Prism 7.0e. Mouse genome (mm9) is available on UCSC Genome Browser (http://genome.ucsc.edu).

For manuscripts utilizing custom algorithms or software that are central to the research but not yet described in published literature, software must be made available to editors and reviewers. We strongly encourage code deposition in a community repository (e.g. GitHub). See the Nature Research guidelines for submitting code & software for further information.

Data

Policy information about availability of data

All manuscripts must include a data availability statement. This statement should provide the following information, where applicable:

- Accession codes, unique identifiers, or web links for publicly available datasets
- A list of figures that have associated raw data
- A description of any restrictions on data availability

Data supporting the findings of this study are available from the authors on reasonable request. The source data underlying Fig. 2b-d, 2g, 3m, Fig. 5g, h, Fig. 6c, Extended Data Fig. 1j, 2b, c, 3g, h, 4h and 5b are provided in Supplemental Table. The single-cell RNA-seq data for this study has been deposited in the Gene

Expression Omnibus under accession number GSE168742 (https://www.ncbi.nlm.nih.gov/geo/query/acc.cgi?acc=GSE168742) and is now available. Single-cell RNA-seq data of cardiomyocytes from p53 knockout mice have been deposited under accession number GSE 95143.

Field-specific reporting

Please select the one below that is the best fit for your research. If you are not sure, read the appropriate sections before making your selection.

☒ Life sciences ☐ Behavioural & social sciences ☐ Ecological, evolutionary & environmental sciences

For a reference copy of the document with all sections, see nature.com/documents/nr-reporting-summary-flat.pdf

Life sciences study design

All studies must disclose on these points even when the disclosure is negative.

Sample size	No statistical methods were used to predetermine sample size estimates. Sample size was determined based on the experimental results that we obtained from preliminary experiments and published papers (https://doi.org/10.1038/s41467-018-06639-7). In vivo studies, we chose standard sample sizes reported in the previous literature of mouse studies. The numbers of performed experiments were indicated in each figure legend.
Data exclusions	Before starting in vivo experiments, we have established the criteria that mice died within 1 week after the operation were excluded from the analysis.
Replication	The number of times each experiment was repeated with similar results is stated in the Methods section. Major experiments were performed with an appropriate sample size to obtain statistical significance. To keep the number of animals used in experiments as low as possible, animal experiments related with TGFb neutralization and AAV9 injection were performed with limited sample size. To make sure that the experiments could be reproduced, at least 2 independent researchers were involved in major experiments (e.g. operation of mice, injection of AAV9, RNA-seq analysis, immunostaining, and smFISH analysis).
Randomization	In vivo experiments, mice were randomly allocated to each group. There was no randomization for in vitro experiments, but the experiments were conducted and checked together by at least 2 independent researchers.
Blinding	Operation and echocardiographic analysis of mice were performed by independent operators who were blinded to genotype of mice. There was no blinding for in vitro cellular experiment. In in vitro experiments, analysis results were confirmed by multiple researchers who did not actually conduct experiments on all raw data.

Reporting for specific materials, systems and methods

We require information from authors about some types of materials, experimental systems and methods used in many studies. Here, indicate whether each material, system or method listed is relevant to your study. If you are not sure if a list item applies to your research, read the appropriate section before selecting a response.

Materials & experimental systems

n/a	Involved in the study	
☐	☒	Antibodies
☒	☐	Eukaryotic cell lines
☒	☐	Palaeontology and archaeology
☐	☒	Animals and other organisms
☐	☒	Human research participants
☒	☐	Clinical data
☒	☐	Dual use research of concern

Methods

n/a	Involved in the study	
☒	☐	ChIP-seq
☐	☒	Flow cytometry
☒	☐	MRI-based neuroimaging

Antibodies

Antibodies used

All antibodies used in this study are commercially available. Antibody validation was performed by the individual manufacturer and their data are available on the manufacturers' website. Please also see Methods for further description of antibodies, including dilutions used.

anti-pSmad2/3(Ser456/467) antibody (Cell Signaling Technology, #8828, D27F4, Lot:7)
anti-Smad2/3 antibody (Cell Signaling Technology, #8685, D7G7, Lot:4)
anti-Smad3 (phospho S423+S425) antibody (Abcam, ab52903, EP823Y, Lot:GR3268135-3)
anti-Smad3 antibody (Abcam, ab40854, EP568Y, Lot:GR3255567-2)
anti-Collagen1 antibody (Abcam, ab138492, EPR7785, Lot:GR3297275-4)
anti-TGF beta1 antibody (Abcam, ab179695, EPR18163, Lot:GR297331-1)
anti-TGF beta1 antibody (Abcam, ab92486)

horseradish peroxidase (HRP)-linked rabbit polyclonal anti-DDDDK-tag antibody (MBL, PM020-7)
HRP-linked horse anti-rabbit IgG antibody (Cell Signaling Technology, #7074)
HRP-linked horse anti-mouse IgG antibody (Cell Signaling Technology, #7076)
anti-TGFβReceptor II antibody (Sigma-Aldrich, SAB4502960)
anti-TGF beta3 antibody (Abcam, ab53727)
anti-Phospho-Histone H2A.X (Ser139) Monoclonal antibody (Invitrogen, #MA1-2022, 3F2, Lot:3043548)
anti-NADPH oxidase 4 antibody (Abcam, ab109225, UOTR1B492, Lot:GR189791-15)
anti-Actin Monoclonal Antibody (Invitrogen, #MA5-11869, ACTN05(C4), Lot:UE2766973A)
anti-rabbit IgG-Alexa 594 (Invitrogen, #A-11037)
anti-mouse IgG-Alexa 647 (Invitrogen, #A-21236)
anti-pSmad3(Ser423/425) antibody (Millipore, #07-1389)
anti-p21 antibody (Abcam, ab188224, EPR18021, Lot:GR3289187-1)
anti-PDGFRa antibody (Cell Signaling Technology, #3174, D1E1E, Lot:GR3195467-5)
isotype IgG1 control antibody (R&D Systems, #MAB002, Lot:TS017051)
anti-TGFβ1 antibody (R&D Systems, MAB240, #9016)
APC anti-mouse CD140a Antibody (BioLegend, #135907, APA5, Lot:B348995)
FITC anti-mouse CD31 Antibody (BioLegend #102506, MEC13.3, Lot:B190608)
anti-HtrA3 antibody (Novus, #NB600-1151)
anti-cardiac troponin I (Abcam, #ab47003)

Validation

All antibodies used in this study were obtained from commercial sources and validated according to manufactures' instruction. The details of manufactures' validation as well as the number of citation by publications can be found on the website of each manufacture. Immunostaining of samples in this study was also compared to unstained or secondary antibody-only controls processed in the same way.

Animals and other organisms

Policy information about studies involving animals; ARRIVE guidelines recommended for reporting animal research

Laboratory animals

Mice were housed in a specific pathogen-free facility with a 12-h light/12-h dark cycle. Ambient room temperature was regulated at 73±5 °F and humidity was controlled at 50±10 %. C57BL/6 were purchased from CLEA JAPAN. Htra3 knockout mice were generated in our laboratory as indicated in the method. Age of mice operated and used for experiments were 9-11 week old male mice.

Wild animals

The study did not involve wild animals.

Field-collected samples

The study did not involve field collected samples.

Ethics oversight

The University of Tokyo Ethics Committee for Animal Experiments

Note that full information on the approval of the study protocol must also be provided in the manuscript.

Human research participants

Policy information about studies involving human research participants

Population characteristics

In The University of Tokyo, heart tissues were obtained immediately after death due to non-cardiac cause (2 control subjects with normal cardiac function) or during left ventricular assist device surgery or heart transplantation (22 patients with heart failure). The population including control subjects and heart failure patients contained 17 males and 7 females, and the mean age at the isolation of cardiomyocytes for scRNA-seq was 46.4 ± 12.2 years.

Recruitment

The recruitments were performed by collecting samples during surgical procedures and autopsies without any bias.

Ethics oversight

All experiments were approved by the ethics committee of the University of Tokyo (G-10032).

Note that full information on the approval of the study protocol must also be provided in the manuscript.

Flow Cytometry

Plots

Confirm that:

- [x] The axis labels state the marker and fluorochrome used (e.g. CD4-FITC).
- [x] The axis scales are clearly visible. Include numbers along axes only for bottom left plot of group (a 'group' is an analysis of identical markers).
- [x] All plots are contour plots with outliers or pseudocolor plots.
- [x] A numerical value for number of cells or percentage (with statistics) is provided.

Methodology

Sample preparation

For the isolation and collection of non-cardiomyocytes, hearts were minced and enzymatically dissociated using 2 mg/mL

	type 2 collagenase (Worthington), 1 mg/mL dispase (Roche), and 20 U/mL DNAse I (Roche), with 5 cycles of digestion for a total 40 min at 37 °C. After removal of cardiomyocytes through 40-µm cell strainer (Greiner), cells were stained with Zombie Green Fixable Viability Kit (BioLegend) and live cells were collected by fluorescence-activated cell sorting (FACS) using a FACSJazz cell sorter (BD Biosciences).
Instrument	FACSJazz cell sorter (BD Biosciences)
Software	BD FACS sorter software (ver 1.2)
Cell population abundance	All the cells sorted for live non-cardiomyocytes were used for scRNA-seq analysis. For the scRNA-seq of cardiac fibroblasts, PDGFRa positive cells were collected.
Gating strategy	Doublet discrimination was performed by FSC-A vs.FSC-H followed by removal of debris. Live non-cardiomyocytes were then gated according to the results of live/dead cell staining. Cardiac fibroblasts were gated according to the result of PDGFRa staining.

☒ Tick this box to confirm that a figure exemplifying the gating strategy is provided in the Supplementary Information.
