# Supplementary material for: Cardiac fibroblasts regulate the development of heart failure via Htra3-TGF-β-IGFBP7 axis
Source: Nat Commun. 2022 Jun 7;13:3275. doi: 10.1038/s41467-022-30630-y (PMC9174232; doi:10.1038/s41467-022-30630-y)
Supplement: Supplementary file 4 — Description of additional supplementary files [file 41467_2022_30630_MOESM4_ESM.docx]

**Cardiac fibroblasts regulate the development of heart failure**

**via Htra3-TGF-β-IGFBP7 axis**

**Ko, et al.**

**Supplementary data information**

**Supplementary Data 1. The ligand and receptor interaction pairs in the heart**

A list of ligand and receptor (LR) interaction pairs in the heart, which were extracted by integrating cell-type-specific gene modules and the ligand and receptor interaction database (Ramilowski JA. et al. Nat. Commun. 2015). Information on which module are assigned to each LR is also provided.

**Supplementary Data 2. Correlation coefficient with fibroblast module expression**

A list of correlation coefficient between fibroblast module expression and gene expression.
